# A Novel Hybrid Point Defect of Oxygen Vacancy and Phosphorus Doping in TiO_2_ Anode for High-Performance Sodium Ion Capacitor

**DOI:** 10.1007/s40820-022-00912-7

**Published:** 2022-08-02

**Authors:** Daming Chen, Youchun Wu, Zhiquan Huang, Jian Chen

**Affiliations:** School of Materials Science and Engineering, Southeast University, Nanjing, 211189 People’s Republic of China

**Keywords:** Defect engineering, P-dopants, Oxygen vacancy, Conductivity, Sodium ion capacitors

## Abstract

Although sodium ion capacitors (SICs) are considered as one of the most promising electrochemical energy storage devices (organic electrolyte batteries, aqueous batteries and supercapacitor, etc.) due to the combined merits of battery and capacitor, the slow reaction kinetics and low specific capacity of anode materials are the main challenges. Point defects including vacancies and heteroatoms doping have been widely used to improve the kinetics behavior and capacity of anode materials. However, the interaction between vacancies and heteroatoms doping have been seldomly investigated. In this study, a hybrid point defects (HPD) engineering has been proposed to synthesize TiO_2_ with both oxygen vacancies (OVs) and P-dopants (TiO_2_/C-HPD). In comparison with sole OVs or P-doping treatments, the synergistic effects of HPD on its electrical conductivity and sodium storage performance have been clarified through the density functional theory calculation and sodium storage characterization. As expected, the kinetics and electronic conductivity of TiO_2_/C-HPD3 are significantly improved, resulting in excellent rate performance and outstanding cycle stability. Moreover, the SICs assembled from TiO_2_/C-HPD3 anode and nitrogen-doped porous carbon cathode show outstanding power/energy density, ultra-long life with good capacity retention. This work provides a novel point defect engineering perspective for the development of high-performance SICs electrode materials.

**Supplementary Information:**

The online version contains supplementary material available at 10.1007/s40820-022-00912-7.

## Highlights

- A novel hybrid point defect design (HPD) with oxygen vacancies and P-dopant is proposed to solve the insufficient kinetic and low specific capacity problems of TiO_2_.
- Through density functional theory calculation and sodium storage characterization, the synergistic effect of HPD is beneficial to improve the conductivity and sodium storage performance of TiO_2_.
- The prepared sodium ion capacitors show outstanding power/energy density, ultra-long life and good capacity retention.

**Supplementary Information:**

The online version contains supplementary material available at 10.1007/s40820-022-00912-7.

## Introduction

Energy storage devices such as lithium-ion capacitors (LICs), sodium-ion capacitors (SICs), aqueous batteries and supercapacitor have found the limelight due to environmental friendliness, reliable safety and cost-effective [1–5]. In comparison, the SICs have the advantages in natural abundance, low cost and weak solvation energy, which are favorable in large-scale applications [6–9]. Different from sodium-ion batteries (SIBs) through the insertion/extraction of sodium ions to achieve energy storage and output, SICs work reversibly under different charge-storage mechanisms, which are coupled with a battery-type anode electrode via insertion/extraction of sodium ions and a capacitor-type cathode electrode via surface adsorption/desorption [8]. However, the main challenge lies in the unsatisfactory kinetic behavior of the anode material when is assembled with the cathode material (typically active carbon, AC) [10, 11]. For example, titanium dioxide (TiO_2_) is considered as an ideal anode material due to its low cost, good cycle stability, high theoretical capacity (335 mAh g^−1^), non-toxicity and nearly zero-strain during charging and discharging process [12–15]. Nevertheless, the low conductivity (~ 10^–12^ S cm^−1^) and the slow diffusion rate of Na^+^ in TiO_2_ severely limit its practical application. Therefore, the development of suitable anode materials is an urgent goal for high-performance SICs.

Point defects engineering (vacancies and heteroatoms doping) in compounds is a popular way to modulate their electronic structure and chemical properties due to the lattice distortion and electron redistribution [16]. For instance, the introduction of oxygen vacancies (OVs) into TiO_2_ can reduce the energy band gap and increase the density of states (DOS), thereby enhancing their electrical conductivity and kinetic [17–19]. In addition, surface functionalization of TiO_2_ by phosphating can stimulate high chemical reaction activity, improve ion diffusion dynamics and electrical conductivity to achieve fast and efficient charge transfer, resulting in better electrochemical performance [20–22]. Similar effects have also been reported by doping other heteroatoms (such as N, S, and F) in TiO_2_ [23–26]. Although the OVs and heteroatoms doping effects have been widely recognized, they still have problems, especially for a high content defect. For example, foreign heteroatoms doping content is usually low, making it difficult to enter the stable TiO_2_ crystal structure [27]. Moreover, some theoretical calculations and experimental results indicate that high amounts of OVs [28, 29] or dopants [30] can reduce the phase stability, which will decrease the diffusion rate of Li^+^, the capacity and cycling performance. Therefore, a rational design of the point defects is still a challenging task.

Recently, some studies have found that the interaction between OVs and heteroatoms can alleviate the bottleneck of single point defect regulation [25, 31]. For example, Fang et al*.* reported a M-TiO_2_@rGO foam with rich OVs and F-doping, which can effectively improve electrons/ions conductivity [32]. It is reasonable to assume that more complicated effects can be generated when the OVs and P dopants are modulated simultaneously for TiO_2_, but yet has rarely been reported. Herein, the effects of OVs, P-doping, and the combined OVs plus P-doping (hybrid point defects, HPD) on the chemical states, crystal structure, conductivities and sodium storage performance of a TiO_2_/C anode derived from Ti-MOFs were investigated systematically by combining density functional theory (DFT) theoretical calculation and experimental characterizations. All the results indicate the superior of HPD in improving the kinetics and reducing the energy barrier of TiO_2_. When paired with nitrogen-doped porous carbon cathode and assembled into hybrid SICs, an extraordinary high energy density of 147.9 Wh kg^−1^ at 360.0 W kg^−1^ can be obtained. Even at higher power density of 18.0 KW kg^−1^, the energy density still reaches 76.8 Wh kg^−1^. Furthermore, the SICs also exhibit an ultra-long life of more than 8000 cycles at 2 A g^−1^ with a high capacity retention of 83.2%, which endow them with great potentials in energy storage applications.

## Experimental

### Sample Synthesis

The TiO_2_/C was firstly obtained using a Ti-MOF precursor according to references [17, 33] through pyrolysis at 600 °C under Ar for 2 h with a heating rate of 2 °C min^−1^. Then, the oxygen vacancies (OVs) containing TiO_2_/C samples were obtained in Ar/H_2_ gas at 500 °C for 1, 2, 3, 4, and 5 h, and named as TiO_2_/C-O1, TiO_2_/C-O2, TiO_2_/C-O3, TiO_2_/C-O4, and TiO_2_/C-O5, respectively. During phosphating, the sample was placed at the downstream of the furnace with sodium hypophosphite (NaH_2_PO_2_) (the mass ratio of TiO_2_/C and NaH_2_PO_2_ was 1:10) at the upstream of the furnace, and then were heated to 450 °C under Ar for 2 h with the heating rate of 5 °C min^−1^. All the TiO_2_/C-O samples were phosphated, and the obtained samples were named as TiO_2_/C-HPD1, TiO_2_/C-HPD2, TiO_2_/C-HPD3, TiO_2_/C-HPD4 and TiO_2_/C-HPD5, respectively. In comparison, the original TiO_2_/C was also treated by phosphating and named as TiO_2_/C-P.

To obtain the nitrogen-doped porous carbon (NPC), the polyacrylonitrile (PAN) was firstly calcined at 800 °C under Ar atmosphere for 2 h with the heating rate of 5 °C min^−1^. Next, the obtained sample was mixed with KOH (the mass ratio of sample and KOH was 1:4) in a mortar and then calcined in Ar atmosphere at 900 °C for 1 h with a heating rate of 5 °C min^−1^. Finally, the obtained sample was washed several times with 1 mol L^−1^ HCl solution until the solution pH = 7. After vacuum drying, nitrogen-doped porous carbon (NPC) was obtained.

### Characterization

The morphology and microstructure of the samples were investigated by scanning electron microscope (SEM, FEI Sirion-200, 10 kV) and transmission electron microscope (TEM, Talos F200X). The crystallographic characteristics of the samples were measured by X-ray diffraction (XRD, Bruker D8-Discover) equipped with a Cu Kα radiation (λ = 1.5418 Å). Raman spectra were measured using a Renishaw Invia (Thermo Fisher) at an excitation laser beam wavelength of 532 nm. The specific surface areas and pore size distribution were performed by Brunauer–Emmett–Teller (BET) nitrogen adsorption–desorption at 77 K via an Autosorb-IQ2 nitrogen adsorption apparatus. X-ray photoelectron spectroscopy (XPS) experiments were carried out on a Thermo Fisher Scientific 250Xi with Al Kα radiation as the X-ray source, and the spectra was referenced to the C 1* s* binding energy of 284.8 eV. The electron paramagnetic resonance (EPR) data was tested by the Bruker A300 in nitrogen test mode, degassing temperature is 200 °C and degassing time is 12 h.

### Electrochemical Measurements

Half-cell test: For the half-cell testing, TiO_2_/C, TiO_2_/C-P, TiO_2_/C-O, TiO_2_/C-HPD, and NPC worked as the working electrodes. Sodium foil was used as the counter electrode, and the glass fiber (Whatman) was used as the separator. The working electrodes were fabricated by mixing the active material (TiO_2_/C, TiO_2_/C-P, TiO_2_/C-O or TiO_2_/C-HPD), carbon black and polyvinylidene difluoride (PDVF) binder in N-methyl-2-pyrrolidone (NMP) with a mass ratio of 8:1:1. The prepared slurry was uniformly coated onto a copper foil and dried at 100 °C for 24 h under vacuum. The NPC electrode was prepared by the same method using NPC as the active material and aluminum foils as the current collector. Afterward, these electrodes were punched into a 12 mm diameter disk with a mass loading of 1.0–2.0 mg per electrode. The coin cells (CR2032-type) were assembled in an Ar-filled glove box, and the electrolyte was a solution of 1 M NaClO_4_ in EC: DEC (v/v 1:1) with 5% FEC.

SICs Devices: The SICs were assembled in coin cells using pre-cycled TiO_2_/C-HPD3 electrode (cycling three times at 50 mA g^−1^ in a half-cell) as anode and NPC as cathode with the mass ratio of 2:1, 1:1 and 1:2. The separator and electrolyte are the same as the Na-ion half-cell.

Electrochemical Measurement: All electrochemical tests were performed at room temperature. The galvanostatic charge/discharge (GCD) measurements were conducted by using a LAND CT2001A battery test system. The cyclic voltammetry (CV) and electrochemical impedance spectra (EIS) measurements were performed on the CHI-660 electrochemical workstation (Chenhua, Shanghai, China). The energy density $E$ (Wh Kg^−1^) and power density $P$ (W Kg^−1^) of SICs can be obtained from the previous working formula [34].

### Computational Details

The Vienna ab initio simulation package (VASP) of DFT was used for theoretical calculations [35]. The exchange correlation function was processed within the generalized gradient approximation (GGA) of the Perdew–Burke–Ernzerhof type (PBE) with a plane wave cut-off energy of 400 eV. All geometry optimizations and electronic structure calculations were calculated using the periodic boundary conditions, and the Brillouin-zone integrations were performed using a 7 × 7 × 7 Monkhorst–Pack grid. The energy convergence criterion and force were set as 10^–5^ eV and 0.01 eV Å^−1^, respectively. A 2 × 2 × 1 supercell containing 16 Ti atoms and 32 O atoms was used for simulation calculation. The changes of the DOS and adsorption energy were calculated, including the original structure, OVs, P-doping and OVs plus P-doping, respectively.

## Results and Discussion

### Microstructure and Composition Analysis

**Fig. 1:**
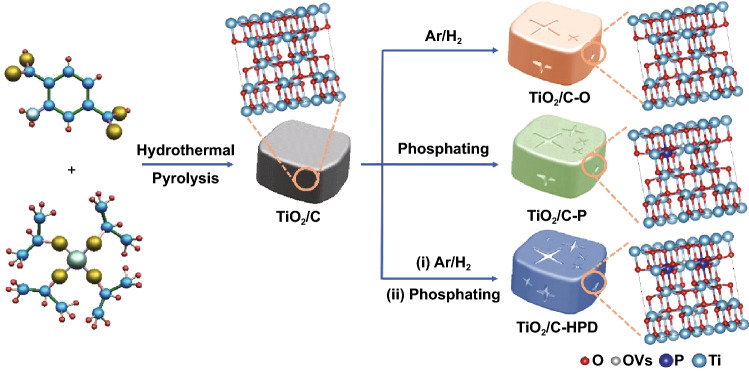
Schematic illustration for the synthesis of the TiO_2_/C, TiO_2_/C-O, TiO_2_/C-P, TiO_2_/C-HPD

Figure 1 shows a schematic diagram of the synthesis for the TiO_2_/C, TiO_2_/C-O, TiO_2_/C-P, and TiO_2_/C-HPD. Succinctly, Ti-MOF can be obtained by facile hydrothermal method using titanium isopropoxide and 2-aminoterephthalic acid. Figure S1 shows that Ti-MOF presents a typical square-like with a diameter of ~ 500 nm. Then, the Ti-MOF was heated at 600 °C for 2 h to obtain the TiO_2_/C with similar morphology and particle size (Fig. S2a). It is well known that reducing hydrogen can react with lattice oxygen on the surface of TiO_2_ to generate Ti^3+^ sites and OVs [26]. Hence, TiO_2_/C-O with different OVs can be obtained by regulating the Ar/H_2_ treatment time. TiO_2_/C-P and TiO_2_/C-HPD can be prepared through phosphating treatment of TiO_2_/C and TiO_2_/C-O, respectively.

**Fig. 2:**
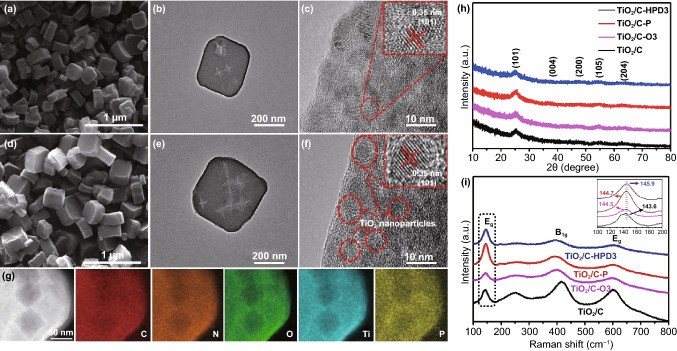
**a**–**c** SEM, TEM and HRTEM images of the TiO_2_/C-O3. **d**–**f** SEM, TEM and HRTEM images of the TiO_2_/C-HPD3. **g** HAADF-STEM image of the TiO_2_/C-HPD3 and the elemental distribution of C, N, O, Ti, and P. **h** XRD pattern and **i** Raman spectra of the TiO_2_/C, TiO_2_/C-P, TiO_2_/C-O3 and TiO_2_/C-HPD3

Compared with pristine TiO_2_/C, the morphologies of TiO_2_/C-O3 (Fig. 2a), TiO_2_/C-HPD3 (Fig. 2d), and TiO_2_/C-P (Fig. S2d) have not change obviously after the introduction of OVs and P atoms into these samples. The TEM images (Figs. 2b, e and S2b, e) prove that the particle sizes are also unaltered, but irregular pits can be observed on the surface of the thermally treated samples. For the TiO_2_/C-HPD series (Fig. S3), more severe pits appear at longer treatment time, suggesting that these microcracks are caused by thermal stress. The close-up HRTEM images show that TiO_2_ nanoparticles with a diameter of ~ 6 nm are dispersed into the carbon framework in all the TiO_2_/C (Fig. S2c), TiO_2_/C-P (Fig. S2f), TiO_2_/C-O3 (Fig. 2c), and TiO_2_/C-HPD3 (Fig. 2f). The lattice fringe spacing is 0.35 nm, which can be attributed to the (101) plane for the anatase TiO_2_. The TEM elemental mapping (Fig. 2g) confirms that C, N, O, Ti, and P elements are evenly distributed in TiO_2_/C-HPD3. There are lower signal in the irregular pits, in agreement of the thermal effects. The BET analysis (Fig. S4) shows that the specific surface areas of TiO_2_/C, TiO_2_/C-P, TiO_2_/C-O3, and TiO_2_/C-HPD3 are 28.71, 56.37, 62.21, and 83.03 m^2^ g^−1^, respectively, in agreement with the finding that the longer exposure at high temperature, the higher surface area. Moreover, all these samples possess the hierarchical pore structure. These results show that the post heat treatment can bring minimal damage for the overall square-like nanostructure of TiO_2_/C. The increased specific surface area is conducive to improving the contact between the active material and the electrolyte, providing more sites for the sodium storage [31, 36].

Figure 2h compares the typical XRD patterns of the TiO_2_/C, TiO_2_/C-O3, TiO_2_/C-P, and TiO_2_/C-HPD3. All these samples show the broad diffraction peaks at ~ 25° corresponding to the (101) plane of the anatase structure, which is consistent with the TEM analysis [12]. Raman spectra of the samples show the typical modes of TiO_2_ (*E*_g_ at 143.6 and 603.3 cm^−1^, *B*_1g_ at 416.3 cm^−1^) in Fig. 2i [31, 37, 38]. It is worth mentioning that TiO_2_/C-O3, TiO_2_/C-P, and TiO_2_/C-HPD3 have wider characteristic peaks, which is due to the fact that the enhanced conductivity by OVs or P-doping can reduce the peak intensity [35]. In addition, the *E*_g_ peak of TiO_2_/C-HPD3 shifts to higher wavenumbers, which can be caused by the distortion of crystal structure and the decrease in Ti–O bonding symmetry [38–40].

XPS is used to further investigate the surface chemical states and components in the samples. All the samples (Fig. S5) have the same signal peaks of C, N, O, and Ti. Besides, the P peaks are detected in the P-doped samples (TiO_2_/C-P, TiO_2_/C-HPD1, TiO_2_/C-HPD3, and TiO_2_/C-HPD5), and the calculated content is listed in Table S1.

**Fig. 3:**
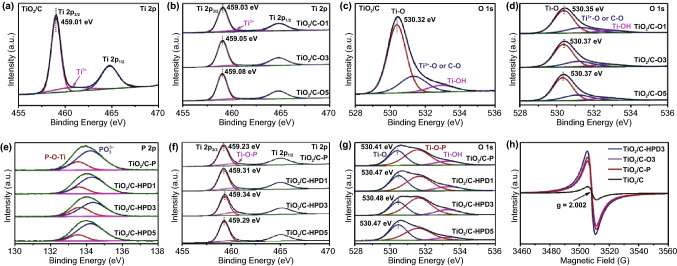
High-resolution XPS spectra: **a**, **b**, **f** Ti 2*p*, **c**, **d**, **g** O 1*s* and **e** P 2*p*. **h** Electron paramagnetic resonance (EPR) spectra of the TiO_2_/C, TiO_2_/C-P, TiO_2_/C-O3 and TiO_2_/C-HPD3

Figure 3a shows the Ti 2*p* spectrum of TiO_2_/C, revealing two strong peaks at 459.01 and 464.76 eV, corresponding to Ti 2*p*_3/2_ and Ti 2*p*_1/2_, respectively. For the TiO_2_/C-O samples (Fig. 3b), these two peaks slightly shift to higher binding energy. Meanwhile, a weak peak at ~ 460.80 eV assigned to Ti^3+^ species [41] can be recognized in all TiO_2_/C and TiO_2_/C-O samples. The peak evolution can be attributed to the introduction of OVs on the surface of TiO_2_ by Ar/H_2_ treatment that reduce the electron density of Ti^4+^ [42, 43]. In terms of the O 1*s* spectra (Fig. 3c), three peaks at 530.32, 531.13, and 532.78 eV are detected for the original TiO_2_/C, which can be assigned to Ti–O, C-O and Ti–OH bonds [44]. However, the C-O peak (typical 531.5 eV) shifts to a lower binding energy, indicating the presence of Ti^3+^-O bonds [24, 45]. After Ar/H_2_ treatments, the TiO_2_/C-O samples (Fig. 3d) shows that the Ti–O peaks slightly shift toward higher binding energy, as well as the enlarged peak area ratio of Ti^3+^-O, indicating the formation of OVs [45]. By analyzing and contrasting the TiO_2_/C samples with different Ar/H_2_ treating time (TiO_2_/C, TiO_2_/C-O1, TiO_2_/C-O3, and TiO_2_/C-O5) in Table S1, the contents of Ti^3+^ and OVs slightly rise with the processing time.

After phosphating, the new P 2*p* spectra can be observed in the TiO_2_/C-P, TiO_2_/C-HPD1, TiO_2_/C-HPD3 and TiO_2_/C-HPD5 (Fig. 3e), which can be decomposed into two peaks at 133.81 and 134.51 eV, corresponding to P-O-Ti and PO_3_^3−^, respectively [22, 46], indicating P can be doped in the cation site by replacing Ti^3+^. Comparing with the TiO_2_/C-P, the TiO_2_/C-HPD1 and TiO_2_/C-HPD3 samples have higher P-O-Ti content, proving the beneficial effects of OVs for the P-doping. The lower P-O-Ti content for TiO_2_/C-HPD5 seems contradictory, but this phenomena can be attributed to the damaged TiO_2_ phase structure caused by massive OVs [29, 47]. In turn, appropriate amounts of OVs are critical to obtain high P-doping content. Furthermore, the Ti 2*p* peaks and O 1*s* peaks for the P-doped samples are analyzed (Fig. 3f-g), respectively. The two main peaks (Ti 2*p*_3/2_ and Ti 2*p*_1/2_) after phosphating are similar to those before phosphating, while the peak area at ~ 460.40 eV exhibits slightly similar trend as the P-O-Ti, indicating that it comes mainly from Ti–O-P rather than Ti^3+^ [35, 46]. More strikingly, the peak at 531.62 eV in O 1*s* for all the P-doped samples (Fig. 3g) is significantly intensified with the same trend as P-O-Ti bonds [48], as shown in Table S1. All the data shows that P elements can replace Ti^3+^ by the formation of P-O-Ti bonds, and compared with TiO_2_/C-P without the previous Ar/H_2_ processing, the P content for TiO_2_/C-HPD3 is increased by ~ 36% due to the addition of ~ 2 at% OVs.

Electron paramagnetic resonance (EPR) measurements are also carried out to investigate the variation of OVs. TiO_2_/C, TiO_2_/C-P, TiO_2_/C-O3, and TiO_2_/C-HPD3 (Fig. 3h) all have signals at g = 2.003 (g-factor), which is due to the unpaired electrons trapped by OVs [31]. Clearly, TiO_2_/C-HPD3 has the highest concentration of OVs, indicating that phosphating after Ar/H_2_ treatment can further increase the concentration of OVs.

### Theoretic Prediction

**Fig. 4:**
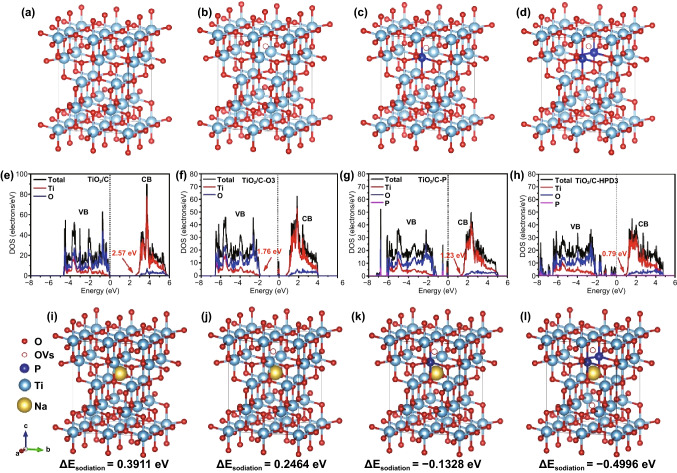
**a-d** The structural models, **e–h** density of states (DOS) analyses, **i-l** Na ion intercalation model of TiO_2_/C, TiO_2_/C-O3, TiO_2_/C-P, and TiO_2_/C-HPD3

As discussed above, various chemical states of P, OVs, and Ti^3+^ have been successfully produced in TiO_2_ through different processing sequences. In order to deeply understand the potential influence of synergistic effects of OVs plus P-doping, the DFT calculation is used to analyze the state of density (DOS) of valence band (VB) and conduction band (CB) of TiO_2_/C, TiO_2_/C-O3, TiO_2_/C-P, and TiO_2_/C-HPD3 samples with their supercell structures (Figs. 4a-d and S6). TiO_2_/C-HPD3 (Fig. 4e-h) has the smallest bandgap (0.79 eV) between its discrete VB and CB due to the HPD, suggesting a better electrical conductivity. In addition, the energy barrier (${\Delta E}_{\mathrm{sodiation}}$) for the sodiation process of TiO_2_ under OVs, P-doping and the HPD is studied by the following formula [25, 49].

1 $$\Delta {\mathrm{E}}_{\mathrm{sodiation}}={\mathrm{E}}_{\mathrm{sodiated}-{\mathrm{TiO}}_{2}}-{\mathrm{E}}_{{\mathrm{TiO}}_{2}}$$

where ${\mathrm{E}}_{\mathrm{sodiated}-{\mathrm{TiO}}_{2}}$, ${\mathrm{E}}_{{\mathrm{TiO}}_{2}}$ and ${E}_{\mathrm{Na}}$ represent the total energy of Na-intercalated TiO_2_, energy of single surface slab of TiO_2_ and Na ions, respectively (Table S2). Figure 4i-l shows the Na ion intercalation models of TiO_2_/C, TiO_2_/C-O3, TiO_2_/C-P, and TiO_2_/C-HPD3 anodes. The calculation results show that TiO_2_/C-HPD3 also has the lowest energy barrier (${\Delta E}_{\mathrm{sodiation}}$=-0.4996 eV), indicating that the HPD is helpful to the insertion of Na ions into TiO_2_. Therefore, the introduction of HPD into TiO_2_ can theoretically improve its kinetics and adsorption energy for Na^+^, which is beneficial to the improvement of electrochemical performance.

### Na-ion Storage Characteristics of TiO_2_/C-HPD

**Fig. 5:**
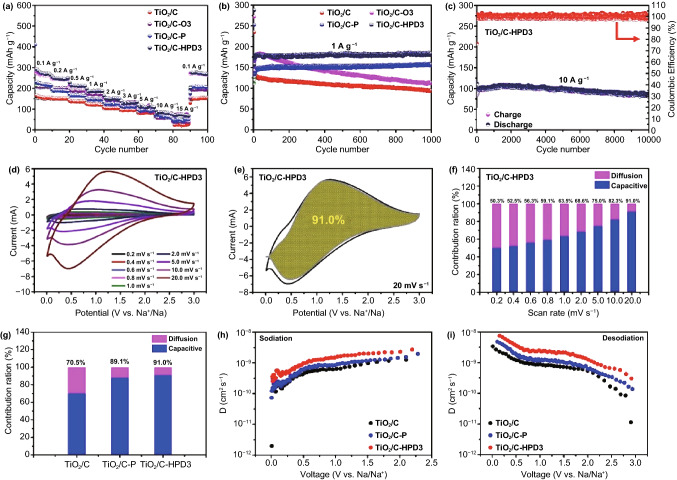
TiO_2_/C, TiO_2_/C-O3, TiO_2_/C-P, and TiO_2_/C-HPD3: **a** Rate performances and **b** cycling performances at a current density of 1 A g^−1^. TiO_2_/C-HPD3: **c** Long-term cycling performance at a current density of 10 A g^−1^, **d** CV curves at various sweep rates, **e** CV curve with capacitive- and diffusion-controlled contributions at 20 mV s^−1^ and **f** Ratio of capacitive contribution at different scan. TiO_2_/C, TiO_2_/C-P and TiO_2_/C-HPD3: **g** Ratio of capacitive contribution at 20 mV s^−1^, Diffusion coefficients calculated from the GITT potential profiles as a function of potential during **h** sodiation and **i** desodiation

The sodium storage properties of all samples are evaluated by using CR2032 coin cells. Figure 5a shows the rate performance of TiO_2_/C, TiO_2_/C-O3, TiO_2_/C-P, and TiO_2_/C-HPD3 samples at various current densities. Strikingly, TiO_2_/C-HPD3 delivers reversible charge capacities of 261.7, 243.5, 213.7, 187.6, 149.3, 134.6, 110.3, and 92.4 mAh g^−1^ at 0.1, 0.2, 0.5, 1, 2, 3, 5, and 10 A g^−1^, respectively. Even at an ultrahigh current density of 15 A g^−1^, its reversible capacity still remains at 82.5 mAh g^−1^. When the current density returns to 0.1 A g^−1^, the reversible capacity can also be restored to 275.3 mAh g^−1^, proving its outstanding tolerance. TiO_2_/C-HPD3 also exhibits the highest specific capacity (183.8 mAh g^−1^) when cycling 1000 cycles at a current density of 1 A g^−1^ (Fig. 5b). These results show that the HPD can improve the electrochemical performance of TiO_2_ and is more effective than the sole OVs or P-doping. In addition, the rate performance and cycling stability of the TiO_2_/C-O and TiO_2_/C-HPD series are also compared (Fig. S7). Among them, TiO_2_/C-O3 has the best electrochemical performance, and thus the TiO_2_/C-O3 is taken as the research object to study the P-doping effects. Furthermore, TiO_2_/C-HPD3 exhibits the best rate performance and cycling stability due to the highest P-doping content through the optimal OVs.

Another advantage of TiO_2_/C-HPD3 is its highly improved cycling durability. As shown in Fig. 5c, it can deliver a decent capacity of 84.1 mAh g^−1^ after 10,000 cycles at 10 A g^−1^, and the capacity retention is as high as 88.6%. Furthermore, the coulomb efficiency of TiO_2_/C-HPD3 is close to 100% during cycles. In addition, TEM images (Fig. S8) show that the morphology and particle size of the TiO_2_/C-HPD3 electrode change very slightly after 500 cycles at 1 A g^−1^, unambiguously supporting its excellent cycle stability and excellent performance. Compared with the Ti-based anode materials in the reported literature, TiO_2_/C-HPD3 also exhibits extraordinary specific capacity, rate performance and cycle stability (Table S3), further demonstrating the promising of TiO_2_/C-HPD3 for Na storage.

To clarify the fast sodium storage mechanism, Fig. S9 shows the CV curve of the TiO_2_/C-HPD3 electrode with a voltage window of 0.01–3 V (vs Na^+^/Na), and the scan rate is 0.2 mV s^−1^. A typical irreversible reaction occurs in the first cycle, which is mainly due to the formation of a solid electrolyte interphase (SEI) layer [50]. The broad cathodic peaks (~ 0.65 V) and anodic peaks (~ 0.8 V) in the next two cycles are attributed to the reversible transformation of Ti^3+^ and Ti^4+^ during Na^+^ intercalation and deintercalation, which was confirmed by Wu et al. via ex-situ XPS [51]. Figures 5d and S10a, d show the CV curves of TiO_2_/C, TiO_2_/C-P, and TiO_2_/C-HPD3 electrodes at different scan rates of 0.2–20 mV s^−1^. In general, the reaction kinetic mechanism can be divided into faradic and non-faradic (pseudo-capacitive) behavior. Therefore, the ratio of the pseudo-capacitive and faradic contribution of peak current (*i*) at different scan rates (*v*) can be calculated by the equation [52].

It can be seen (Fig. S11) that at a scan rate of 0.2–20 mV s^−1^, the peak $b$ values of the cathodic and anodic are 0.89 and 0.93, respectively, indicating that the sodium storage kinetics is controlled by both the pseudo-capacitive and diffusion process. At a scan rate of 20 mV s^−1^, the pseudo-capacitive contribution rate of the TiO_2_/C-HPD3 electrode is as high as 91.0%, which is higher than that of the TiO_2_/C (70.5%) and TiO_2_/C-P (89.1%) electrodes (Figs. 5e, g and S10b, e). Furthermore, Figs. 5f and S10c, f show that the capacitance contribution increases with the increase in scan rate, indicating that pseudo-capacitance is dominant at the high scan rates. The high capacitive behavior of TiO_2_/C-HPD3 is mainly attributed to the synergistic effect between the HPD, which can provide abundant active sites for the storage of Na^+^ and effectively improve the conductivity, thereby enhancing the charge storage capacity of reversible pseudo-capacitance.

The kinetics of these samples are further illustrated by EIS measurement (Fig. S12). Table S4 lists the fitted impedance value, which uses the equivalent circuit model (Fig. S12). By comparing with other electrode materials, it can be known that TiO_2_/C-HPD3 has the smallest values of *R*_s_, *R*_f_, and *R*_ct_ (1.55, 112.6, and 226.5 Ω), indicating that it has the highest conductivity and the fastest kinetics [42]. Clearly, the HPD can effectively improve the conductivity of the material itself. Except for the EIS test, the diffusivity coefficients of sodium ions (D_Na_^+^) of TiO_2_/C, TiO_2_/C-P and TiO_2_/C-HPD3 electrodes are obtained by galvanostatic intermittent titration technique (GITT). TiO_2_/C-HPD3 (Figs. 5h-i and S13) also shows the highest diffusion coefficient (during the sodiation process varies from 9.5 × 10^–9^ to 8.6 × 10^–8^ cm^2^ s^−1^), which further confirms that the HPD is beneficial to improve the electrochemical reaction kinetics. In general, the synergistic effect of HPD can effectively improve the kinetics and conductivity of the material, reduce the energy barrier, and enhance the electrochemical performance, which well confirms by the above DFT and experimental results.

### Electrochemical Performance of TiO_2_/C-HPD3//NPC Hybrid SICs

The nitrogen-doped porous carbon (NPC) through carbonization of PAN and KOH activation is used as the cathode materials for SICs (Fig. S14). The XPS spectrum (Fig. S15a) shows that NPC is composed of C, N, and O elements (87.58, 4.46, and 7.96 at%, respectively), indicating that there are abundant N atom functional groups on its surface. Moreover, the NPC displays a hierarchically porous (Fig. S15b) with an extremely high specific surface area of 2320.7 m^2^ g^−1^.

The NPC electrode is assembled into a half-cell, and its electrochemical performance is evaluated in the potential range of 2.5–4.2 V (vs Na/Na^+^). At different scan rates, the CV curves of NPC electrode is quasi-rectangular with a slight hump, indicating that the double layer behavior is dominant, which is attributed to the material surface defects and heteroatom (N) functional groups (Fig. S16a) [44]. The average discharge capacities of NPC cathode are 98.1, 90.3, 83.3, 77.8, 74.6, 70.8, 68.5, 60.6, and 58.6 mAh g^−1^ at 0.1, 0.2, 0.5, 1, 2, 3, 5, 10 and 15 A g^−1^, respectively (Fig. S16b). When the current density returns to 0.1 A g^−1^, its discharge capacity recovers to 92.9 mAh g^−1^, indicating excellent rate performance. As can be seen from the GCD curves (Fig. S16c), NPC electrode also exhibits a relatively high capacitance of 176.5 F g^−1^ at 0.2 A g^−1^, and remains 138.1 F g^−1^ even at a high current density of 5 A g^−1^. Furthermore, the GCD linear curve has no obvious IR drop at different current densities, indicating that anions (ClO_4_^−^) can be rapidly adsorbed/desorbed on the surface of NPC [44]. Immediately afterward, the NPC cathode (Fig. S16d) is also subjected to a long cycle performance test (90.1 mAh g^−1^ after 2000 cycles at 1 A g^−1^). Compared with commercial AC, NPC has better cycle stability. The excellent electrochemical performance of NPC cathode can be attributed to the following two points: i) Unique hollow nanostructure, which is beneficial to increase the specific surface area and provides sufficient positions for the interaction between the electrode and the electrolyte; ii) Abundant N atoms can improve ion diffusion efficiency [53]. Therefore, if high kinetic TiO_2_/C-HPD3 anode can be coupled with high capacitance NPC cathode, the mismatch of capacitance and kinetic behavior can be minimized, resulting in high-performance SICs [44].

In order to obtain the best TiO_2_/C-HPD3//NPC hybrid SICs, the operating potential window is optimized by testing at different potential ranges (Fig. S17a). When the voltage window is 0–4.2 V, the CV curve deviates from the rectangle with slight polarization, which may be caused by the reaction between the electrode material and the electrolyte. However, when the voltage window changes to 1–4 or 0–4 V, the CV curve is quasi rectangular.

**Fig. 6:**
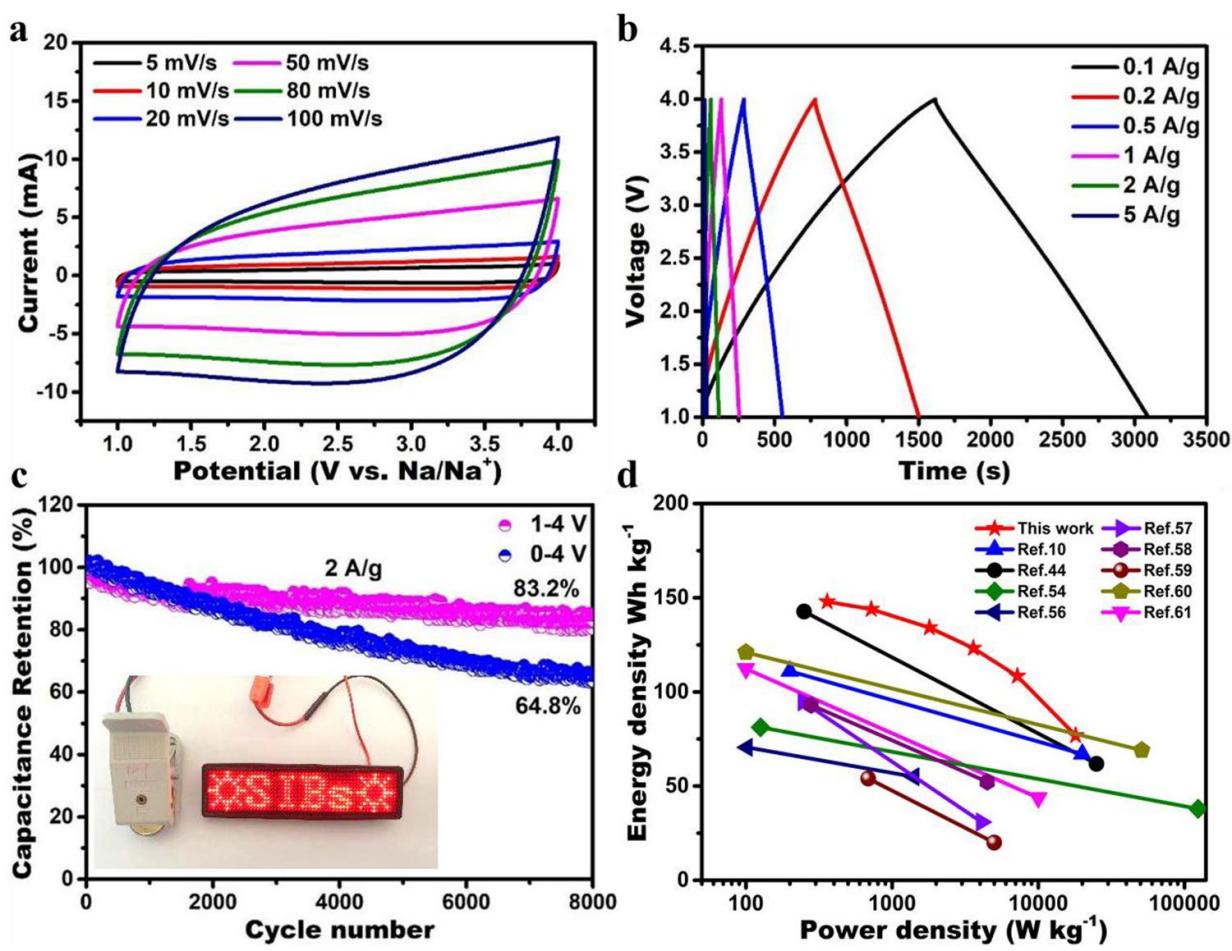
**a** CV curves at different scan rates. **b** Galvanostatic charge/discharge curves. **c** Long-term cycle performance of TiO_2_/C-HPD3//NPC SICs. **d** Ragone plots of this work compared with the reported literature

Figures 6a and S17b show the CV curves of hybrid SICs under two voltage windows with a scanning rate of 5–100 mV s^−1^. All the CV curves show approximately rectangular, indicating that the charge storage mechanism of SICs is a combination of faraday and non-faraday reactions. The GCD curves of the hybrid SICs (Figs. 6b and S17c) are similar to a trend of linear without diffusion limiting process, indicating good reversibility and fast storage dynamics [54]. Furthermore, the hybrid SICs (Fig. 6c) exhibit an ultra-long cycle life with a capacity retention of 83.2% for over 8000 cycles at 2 A g^−1^ (voltage window is 1–4 V). Figure S18 shows the XRD patterns and SEM images of the TiO_2_/CHPD3 electrode before and after cycling. It can be seen that the morphology and crystallinity have not changed significantly, again proving that TiO_2_/CHPD3 has excellent structural stability. However, after different cycles, cracks occur inside the electrode and grow larger and larger. This is probably caused by the internal stress produced by the irreversible volume expansion/shrinkage of the electrode materials during cycling at high current densities, which can cause the active particles to fall off, resulting in the degradation of the battery performance [55]. However, when the voltage window is 0–4 V, the capacity retention rate is only 64.8%. From the above, the best electrochemical window of TiO_2_/C-HPD3//NPC hybrid SICs is 1–4 V. To demonstrate its potential application, the hybrid SICs is used to illuminate the LEDs (as shown in the inset photograph in Fig. 6c).

Meanwhile, the result (Fig. S19) shows that the best mass ratio of the anodic and cathodic materials is 1:1, which is conducive to obtaining the maximum energy density of the SICs device. As expected, Fig. 6d summarizes the energy density and power density of the hybrid SICs, which can be calculated by formulas. Specifically, TiO_2_/C-HPD3//NPC hybrid SICs exhibit the maximum energy density (147.9 Wh kg^−1^) at a power density of 360.0 W kg^−1^. Even at a power density of 18.0 kW kg^−1^, it still provides a decent energy density of 76.8 Wh kg^−1^. Obviously, our work exhibits superior power/energy density compared with recent SICs literatures [10, 44, 54, 56–61]. In addition, self-discharge performance is also a key parameter of energy storage devices [62]. Figure S20 shows the self-discharge process of TiO_2_/C-HPD3//NPC SICs, whose open-circuit voltage can still be maintained at ~ 3.75 V after 24 h, indicating that the fully charged SICs have a very low self-discharge rate.

## Conclusion

In summary, an HPD engineering strategy with oxygen vacancies and P-dopants is proposed to address the poor conductivity and low capacity of TiO_2_ anode. It has been confirmed that the synergistic effect of HPD can effectively improve the electrical conductivity, kinetics and sodium storage performance of TiO_2_/C-HPD3 by density functional theory calculations and electrochemical results. As excepted, TiO_2_/C-HPD3 anode exhibit excellent rate performance (261.7 mAh g^−1^ at 0.1 A g^−1^ and 82.5 mAh g^−1^ at 15 A g^−1^, respectively) and outstanding cycle stability (84.1 mAh g^−1^ at 10 A g^−1^ after 10,000 cycles). Furthermore, the hybrid SICs assembled with TiO_2_/C-HPD3 and nitrogen-doped porous carbon also exhibit excellent energy/power density (360.0 W kg^−1^ at 147.9 Wh kg^−1^ and 18.0 kW kg^−1^ at 76.8 Wh kg^−1^, respectively), ultra-long life (8000 cycles at 2 A g^−1^) with 83.2% of the capacity retention. Importantly, it is hoped that this work can provide a new avenue for the development of high-performance SICs electrode materials.

## Supplementary Information

Below is the link to the electronic supplementary material.

Supplementary file1 (PDF 2305 kb)
