# Supplementary material for: A Novel Hybrid Point Defect of Oxygen Vacancy and Phosphorus Doping in TiO2 Anode for High-Performance Sodium Ion Capacitor
Source: Nanomicro Lett. 2022 Aug 2;14:156. doi: 10.1007/s40820-022-00912-7 (PMC9346024; doi:10.1007/s40820-022-00912-7)
Supplement: Supplementary file 1 — Supplementary file1 (PDF 2305 kb) [file 40820_2022_912_MOESM1_ESM.pdf]

Supporting Information for

A Novel Hybrid Point Defect of Oxygen Vacancy and Phosphorus Doping in TiO_2 Anode for High-Performance Sodium Ion Capacitor

Daming Chen[1], Youchun Wu[1], Zhiquan Huang[1], Jian Chen[1, *]

[1]School of Materials Science and Engineering, Southeast University, Nanjing 211189, P. R. China

*Corresponding author. E-mail: j.chen@seu.edu.cn (J. C.)

Supplementary Figures and Tables

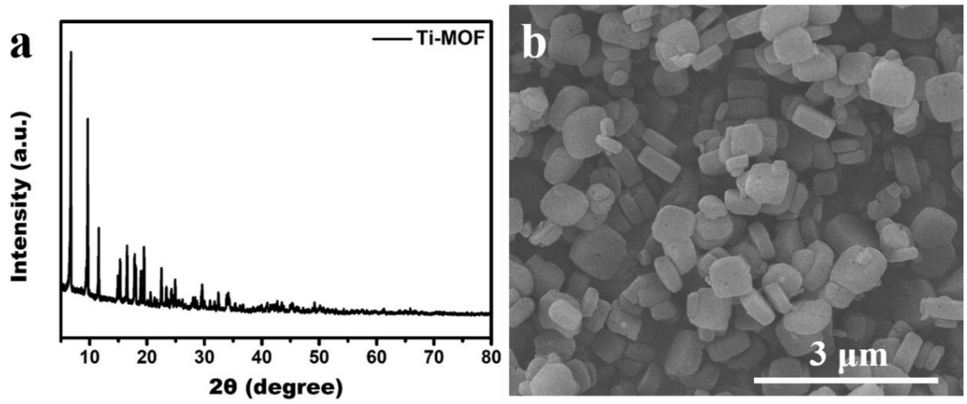

Fig. S1 a, b XRD pattern and SEM image of Ti-MOF

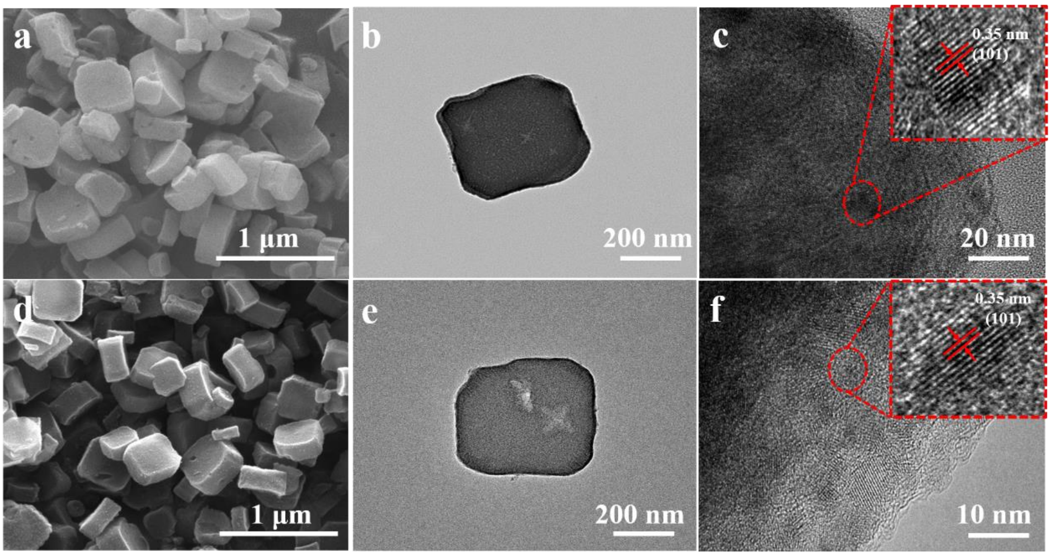

Fig. S2 a SEM image of TiO_2/C. **b** TEM image of TiO_2/C. **c** HRTEM image of TiO_2/C. **d** SEM image of TiO_2/C-P. **e** TEM image of TiO_2/C-P. **f** HRTEM image of TiO_2/C-P

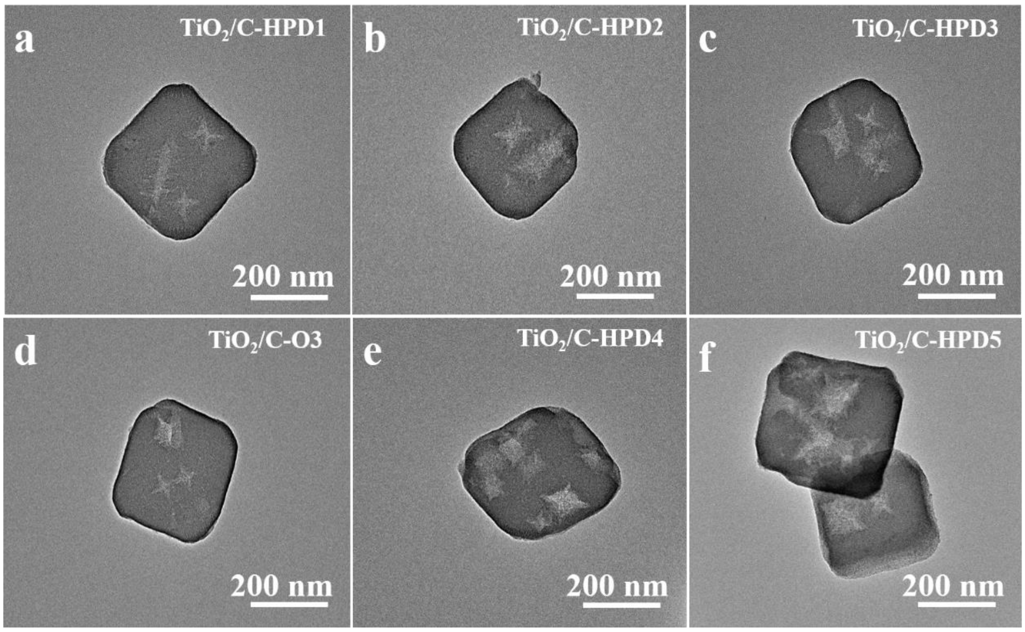

Fig. S3 a-f TEM images of TiO_2/C-HPD1, TiO_2/C-HPD2, TiO_2/C-HPD3, TiO_2/C-O3, TiO_2/C-HPD4 and TiO_2/C-HPD5, respectively

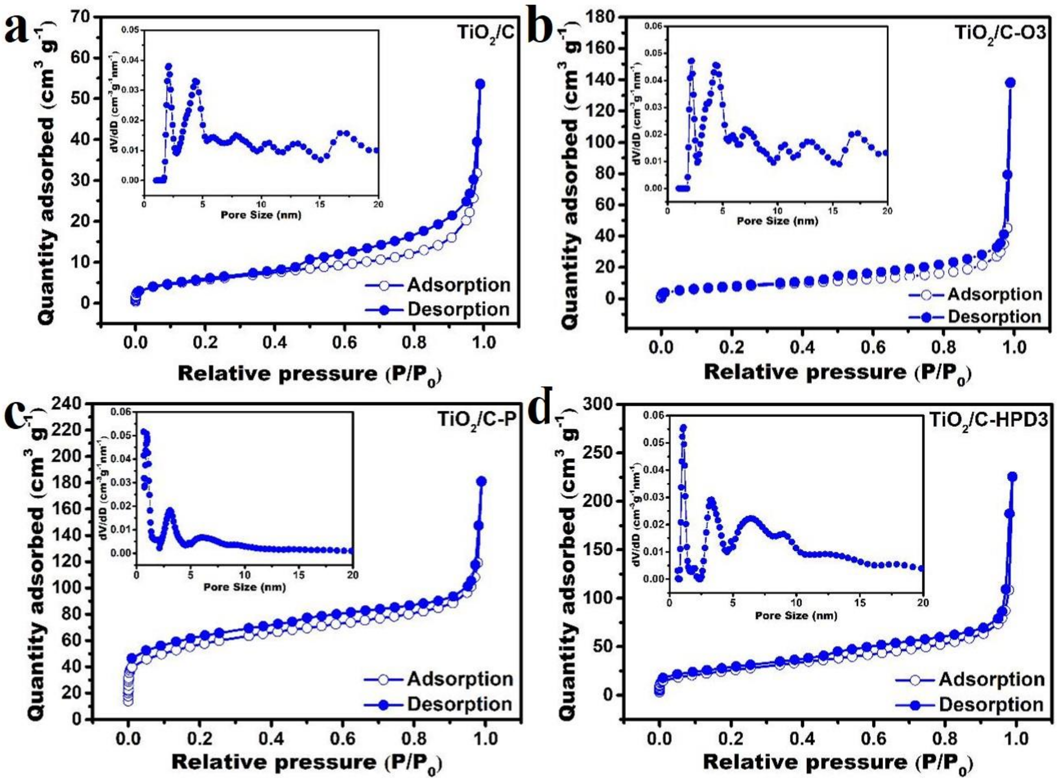

Fig. S4 N_2 adsorption-desorption isotherm and pore size distribution of the TiO_2/C, TiO_2/C-O3, TiO_2/C-P and TiO_2/C-HPD3, respectively

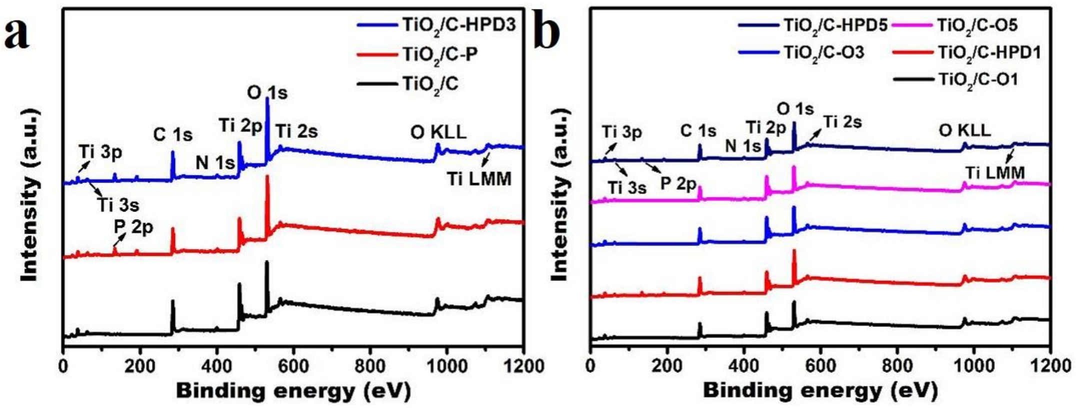

Fig. S5 a, b XPS spectra of the TiO_2/C, TiO_2/C-P, TiO_2/C-HPD1, TiO_2/C-O1, TiO_2/C-HPD3, TiO_2/C-O3, TiO_2/C-HPD5 and TiO_2/C-O5

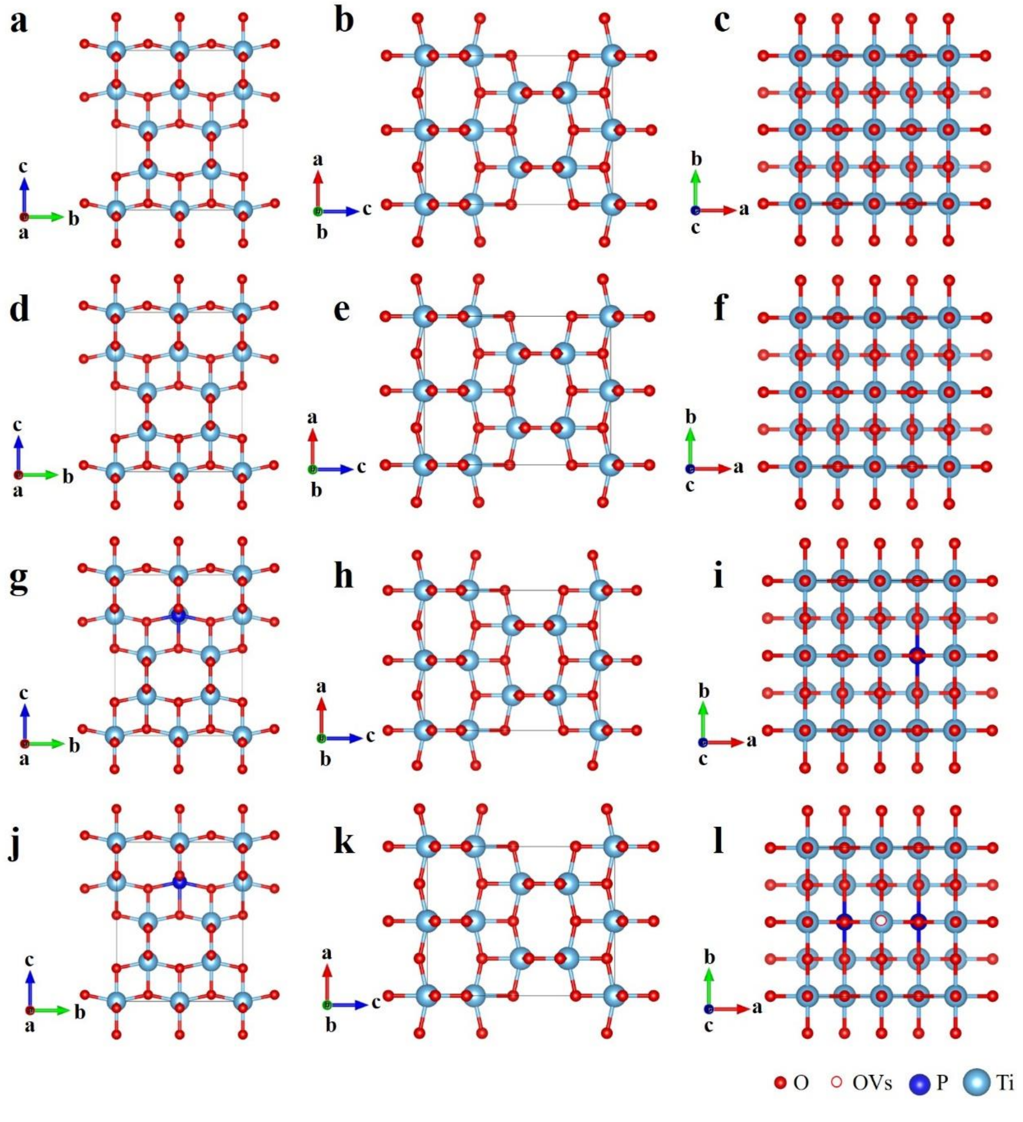

Fig. S6 a-c The structural models of TiO_2/C. **d-f** The structural models of TiO_2/C-O3. **g-i** The structural models of TiO_2/C-P and **j-l** The structural models of TiO_2/C-HPD3

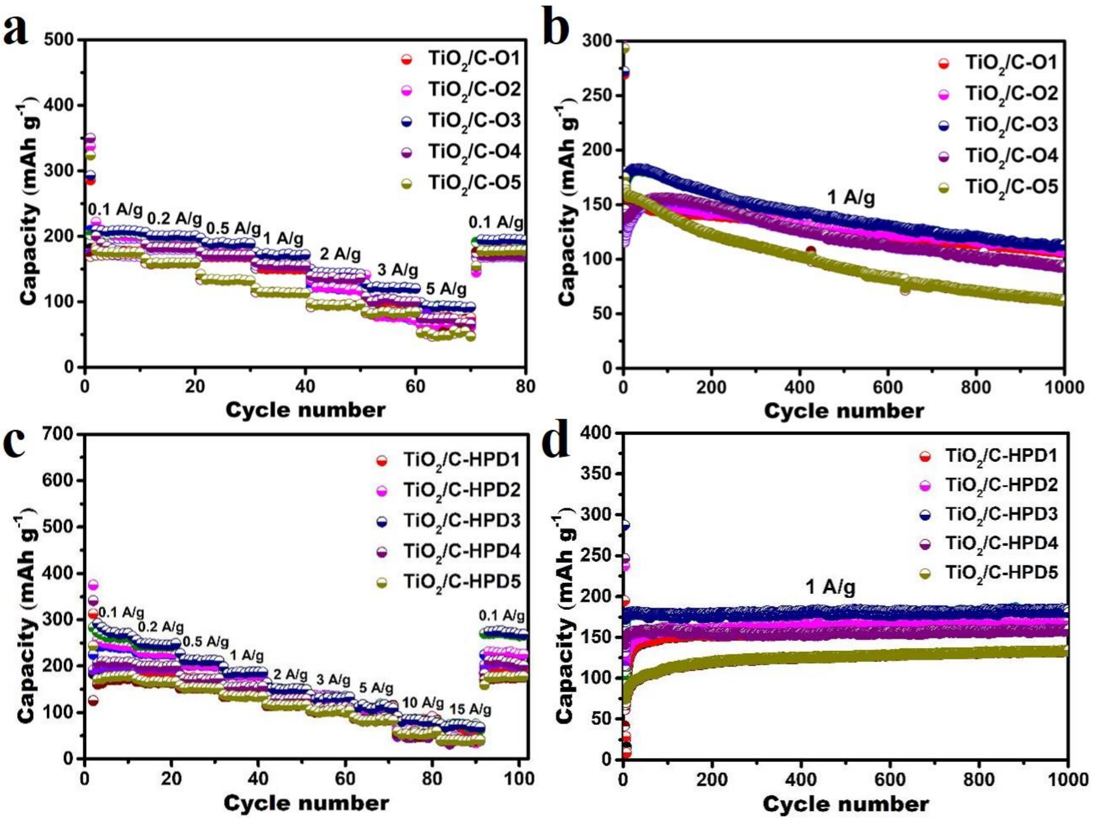

Fig. S7 a, b Rate performances and cycling performances of TiO_2/C-O1, TiO_2/C-O2, TiO_2/C-O3, TiO_2/C-O4 and TiO_2/C-O5. **c, d** Rate performances and cycling performances of TiO_2/C-HPD1, TiO_2/C-HPD2, TiO_2/C-HPD3, TiO_2/C-HPD4 and TiO_2/C-HPD5

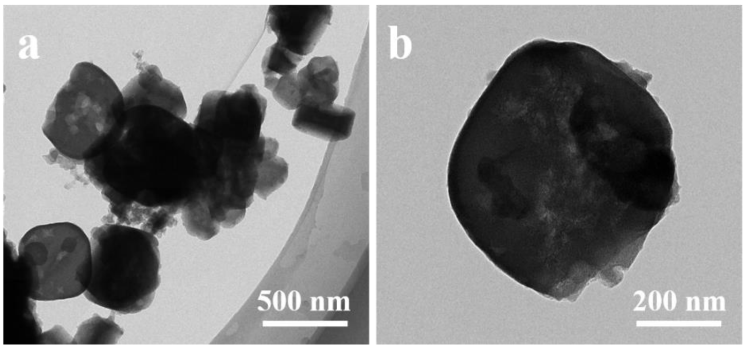

Fig. S8 a, b TEM images of TiO_2/C-HPD3 electrode after 500 cycles at 1 A g^{-1}

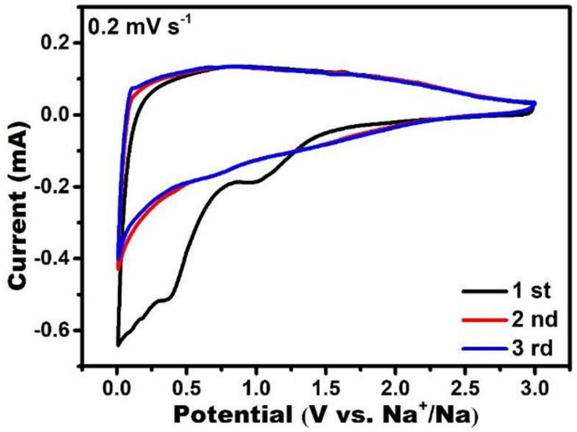

Fig. S9 Typical CV curves of the TiO_2/C-HPD3 at a scan rate of 0.2 mV s^{-1} within 0.01-3.0 V

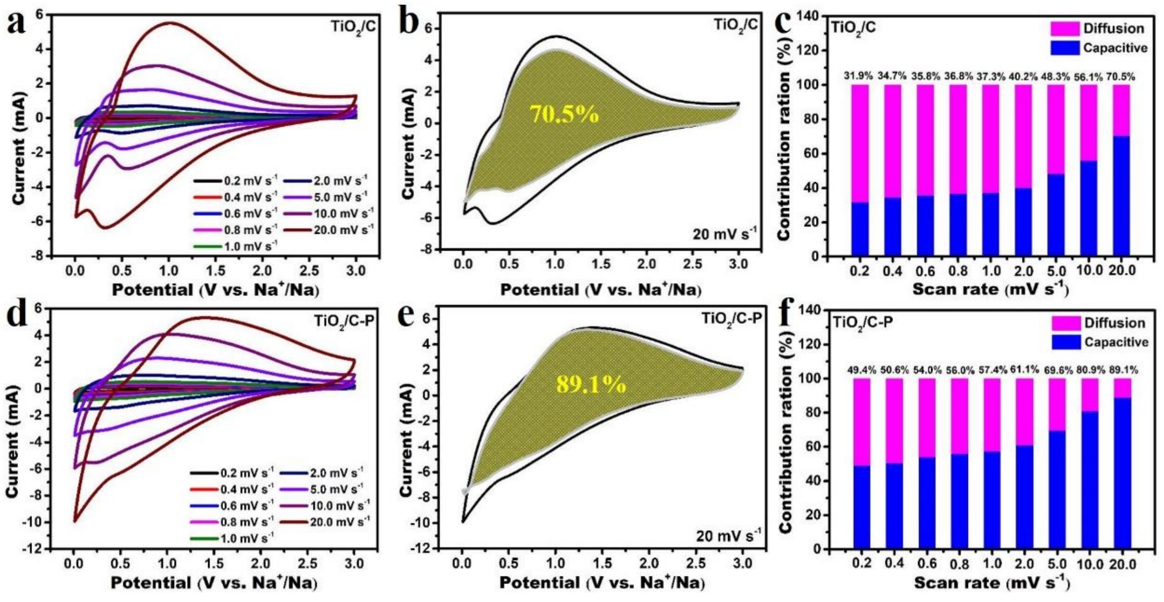

Fig. S10 a CV curves of TiO_2/C at various sweep rates. **b** CV curve with capacitive- and diffusion-controlled contributions at 20 mV s^{-1}. **c** Ratio of capacitive contribution in TiO_2/C at different scan. **d** CV curves of TiO_2/C-P at various sweep rates. **e** CV curve with capacitive- and diffusion-controlled contributions at 20 mV s^{-1}. **f** Ratio of capacitive contribution in TiO_2/C-P at different scan

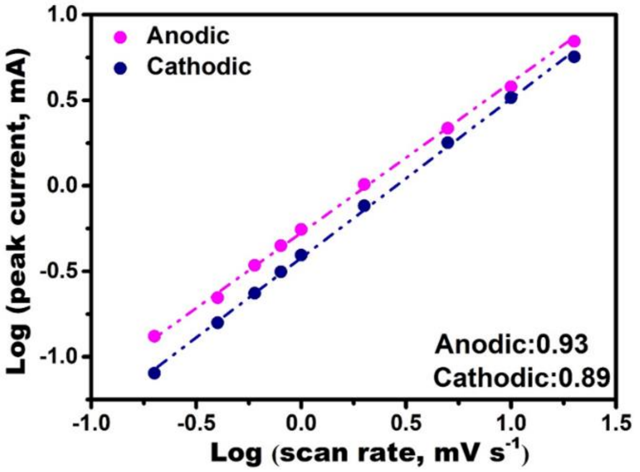

Fig. S11 Log (i) versus log (v) profile of TiO_2/C-HPD3

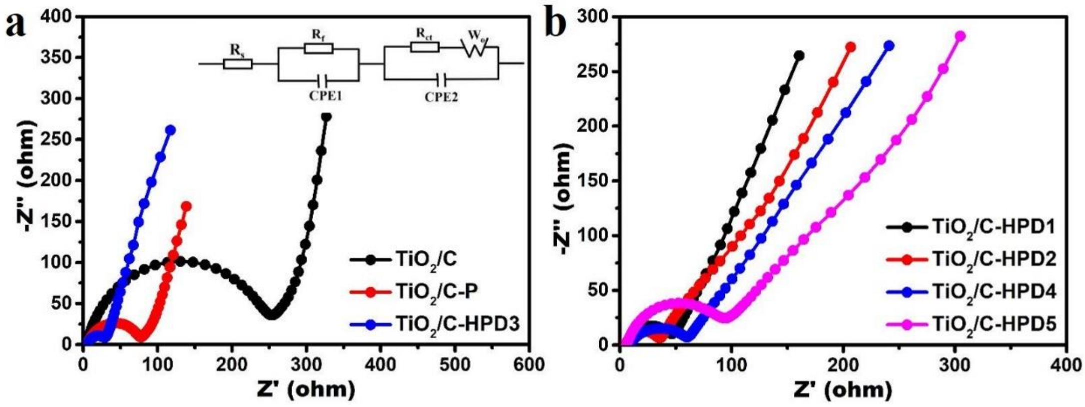

Fig. S12 a, b Nyquist plots of TiO_2/C, TiO_2/C-P, TiO_2/C-HPD1, TiO_2/C-HPD2, TiO_2/C-HPD3, TiO_2/C-HPD4 and TiO_2/C-HPD5; the insert section is the equivalent circuit

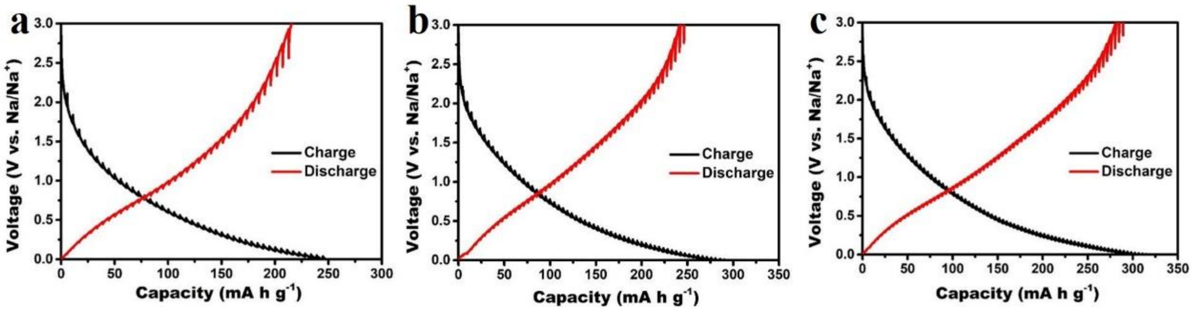

Fig. S13 GITT potential profiles of **a** TiO_2/C, **b** TiO_2/C-P and **c** TiO_2/C-HPD3

The diffusion coefficient (D_{Na^+}) in TiO_2/C, TiO_2/C-P and TiO_2/C-HPD3 electrodes can be calculated from the GITT potential profiles (with a 10 min constant current pulse of 50 mA followed by a relaxation process lasted 30 min) through Fick's second law according to the following equation:

$$D = \frac{4}{\pi\tau}\left(\frac{m_B V_M}{M_B S}\right)^2 \left(\frac{\triangle E_S}{\triangle E_\tau}\right)^2 \qquad \text{(S1)}$$

where τ represents the duration of the current pulse, m_B represents the mass of the active materials, V_M is the molar volume of the samples, M_B represents the molecular weight, S is the total surface electrode in contact with the electrolyte, $\triangle E_S$ denotes the quasi-thermodynamic equilibrium potential difference before and after the current pulse, $\triangle E_\tau$ is the potential difference during current pulse.

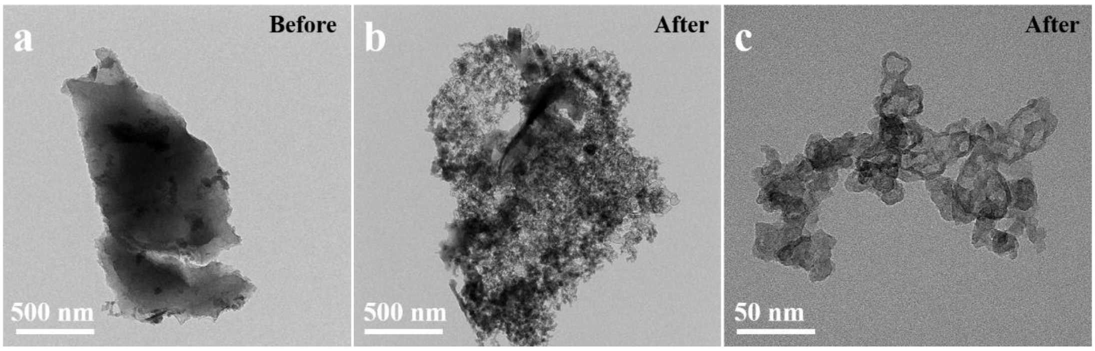

Fig. S14 a-c TEM images of the NPC before and after etching

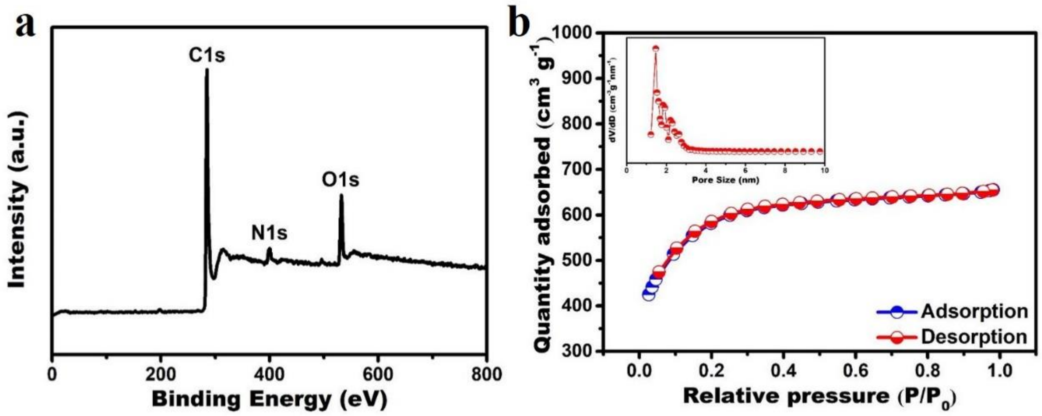

Fig. S15 a XPS spectra of the NPC. **b** N_2 adsorption-desorption isotherm and pore size distribution of the NPC

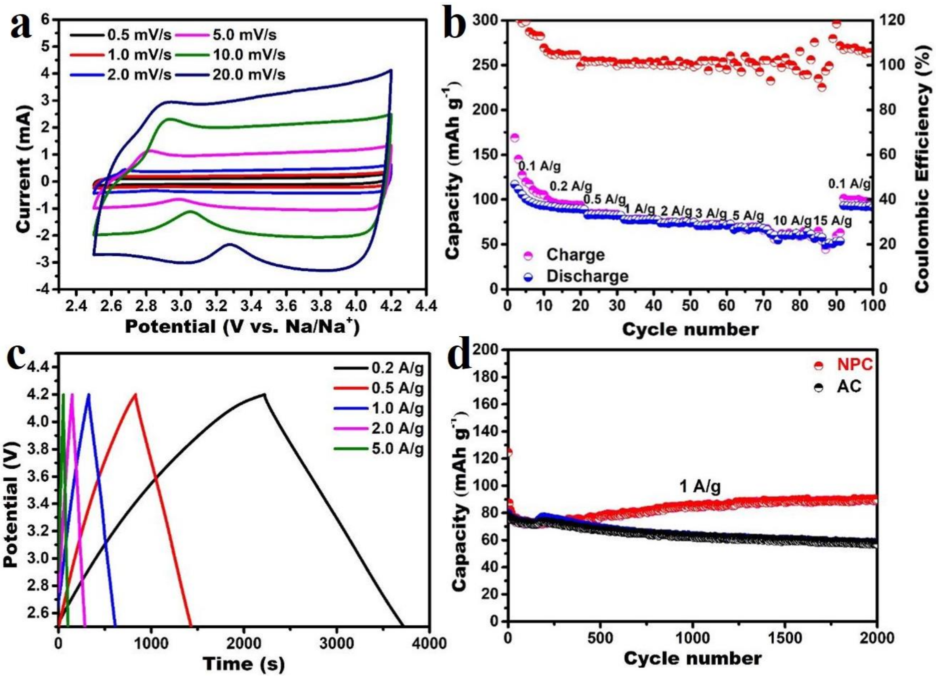

Fig. S16 Electrochemical performance of NPC: **a** CV curves at different scan rates, **b** rate performance, **c** GCD curves and **d** Cycling performances of NPC and AC at a current density of 1 A g^{-1}

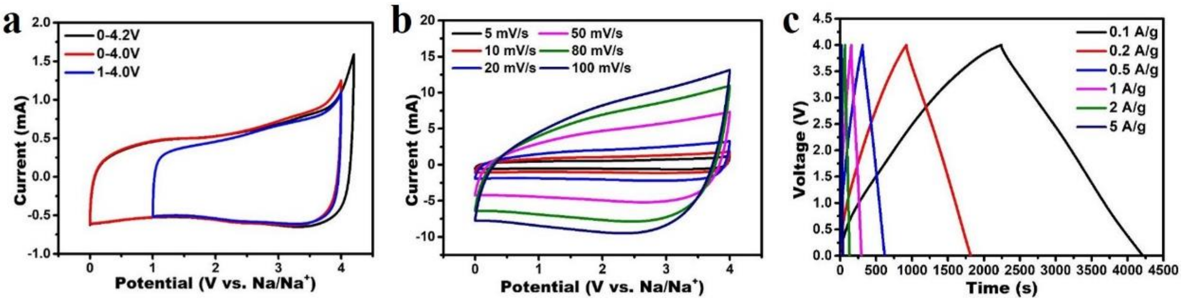

Fig. S17 a CV curves of TiO_2/C-HPD3//NPC SICs at scan rate of 5 mV s^{-1}. **b** CV curves of at different scan rates. **c** Galvanostatic charge/discharge curves

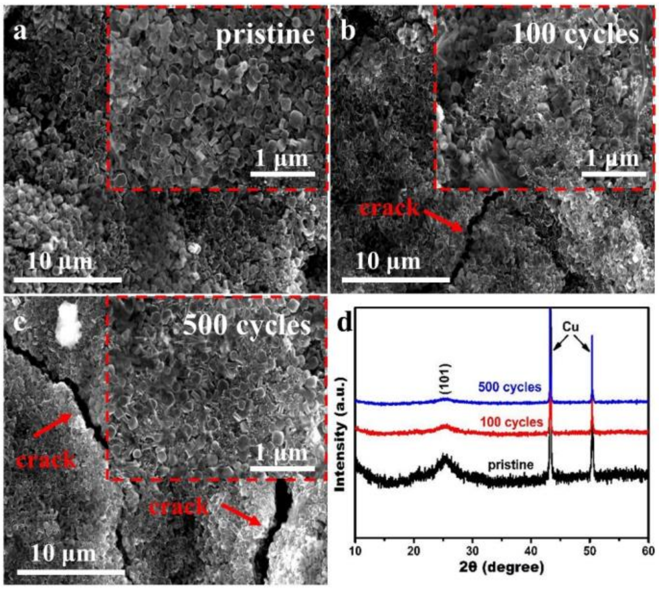

Fig. S18 a-d SEM images and XRD patterns of TiO_2/C-HPD3 electrodes after different cycles of TiO_2/C-HPD3//NPC SICs at a current density of 2 A g^{-1}

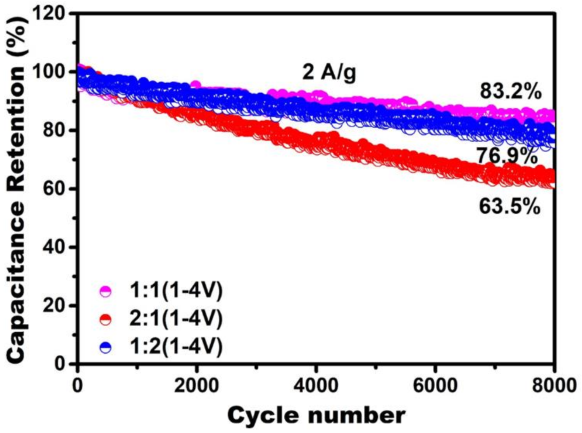

Fig. S19 Long-term cycle performance of TiO_2/C-HPD3//NPC SICs at different mass ratio

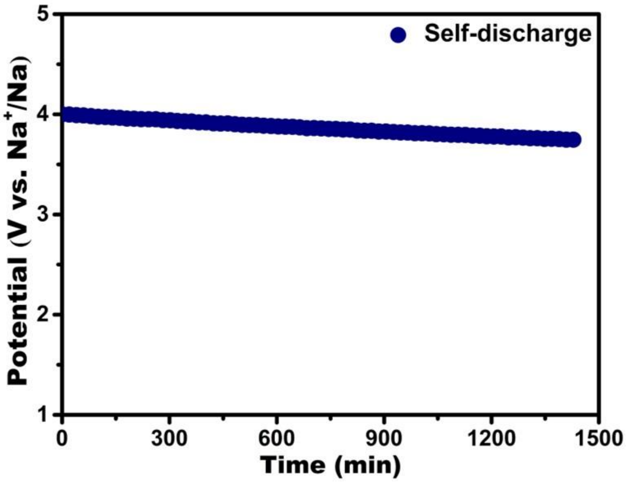

Fig. S20 Self-discharge curves of TiO_2/C-HPD3//NPC SICs after charging to 4V

Table S1 Ti^{3+}, OVs, P, Ti-O-P and P-O-Ti content calculated from XPS results

Species	TiO_2/C	TiO_2/C-O1	TiO_2/C-O3	TiO_2/C-O5	TiO_2/C-P	TiO_2/C-HPD1	TiO_2/C-HPD3	TiO_2/C-HPD5
**Ti^{3+}**	9.05%	10.32%	10.88%	11.38%	-	-	-	-
**OVs**	25.05 %	25.84%	26.94%	29.40%	-	-	-	-
**P**	-	-	-	-	6.75%	8.04%	9.17%	6.47%
**Ti-O-P**	-	-	-	-	13.91%	13.99%	16.39%	12.67%
**P-O-Ti**	-	-	-	-	22.76%	24.68%	33.81%	18.69%

Table S2 Adsorption energy of TiO_2/C, TiO_2/C-O3, TiO_2/C-P and TiO_2/C-HPD3

Sample	Esodiated TiO2/eV	ETiO2/eV	ENa/eV	ΔEsodiation/eV
TiO_2/C	-368.0925	-367.1483	-1.3353	0.3911
TiO_2/C-O3	-368.0925	-367.1483	-1.3353	0.2464
TiO_2/C-P	-356.6367	-355.1686	-1.3353	-0.1328
TiO_2/C-HPD3	-343.7680	-341.9331	-1.3353	-0.4996

Table S3 Comparison of sodium storage performance with reported Ti-based materials

Sample	Current density (A g^{-1})	Cycle number	Specific capacity (mAh g^{-1})	Rate capacity (current density)	Refs.
**TiO_2/C-HPD3**	**0.2**	**300**	**239.4**	**92.4 (10); 82.5 (15)**	**This work**
	**1**	**1000**	**183.8**		
	**10**	**10000**	**84.1**		
**HTTFs-1h**	0.2	200	188.7	113.1 (10)	**[S1]**
	5	10000	132.5		
**TiO_2@$TiOF_2$-30 h**	0.5	2000	151.7	115.4 (5)	**[S2]**
	5	10000	101.2		
**TiS_2/S-TiO_2/C**	0.1	400	274	114.2 (5)	**[S3]**
	3	1500	161		
	10	10000	58		
**TiO_2/C**	0.1	300	206	105 (10)	**[S4]**
	10	10000	-		
**TiO_2-S**	0.1	200	236.3	148.9 (2)	**[S5]**
	1	1000	171.3		
**TiO_2**	0.1	100	207	110 (1)	**[S6]**
**M-TiO_2@rGO**	5	5000	123.3	142 (2)	**[S7]**
**TiO_2(A/B)-MS**	2.5	1000	-	50 (12.5)	**[S8]**
**yolk@shell TiO_{2-x}**	1	1000	99.8	68.6 (5)	**[S9]**
**TiO_2/SCNT**	3.35	1000	118	60 (16.75)	**[S10]**
**S-TiO_2/CS**	10	5000	100.5	120 (6.7)	**[S11]**
**TiO_2-HS**	5	4000	119	112 (12.8)	**[S12]**
**N/S-TiO_2**	0.5	1000	90	75 (1)	**[S13]**
**P-TiO_2**	3.35	1000	141	147 (3.35)	**[S14]**
**TiO_2 nanosheets**	0.5	2500	120	120 (1)	**[S15]**
**TiO_2∩NPCSs**	0.67	3000	152	85 (13.4)	**[S16]**
**TiO_2**	0.5	1400	98	102 (1.5)	**[S17]**
**TiO_2@$Ti_3C_2T_x$**	0.96	5000	110	68 (3.84)	**[S18]**

Table S4 Impedance parameters of the fitting equivalent circuit

Sample	R_s (Ω)	R_f (Ω)	R_{ct} (Ω)
**TiO_2/C**	7.43	265.8	594.8
**TiO_2/C-P**	2.86	152.9	417.0
**TiO_2/C-HPD1**	2.35	141.7	315.8
**TiO_2/C-HPD2**	1.83	128.4	308.4
**TiO_2/C-HPD3**	1.55	112.6	226.5
**TiO_2/C-HPD4**	1.98	135.6	296.0
**TiO_2/C-HPD5**	3.87	181.4	375.1

Supplementary References

[S1] P. Xue, Q. Li, W. Gong, Z. Sun, H. Wang et al., Structure-induced partial phase transformation endows hollow TiO_2/TiN heterostructure fibers stacked with nanosheet arrays with extraordinary sodium storage performance. J. Mater. Chem. A **9**(20), 12109-12118 (2021). https://doi.org/10.1039/d1ta01729b

[S2] S. Guan, Q. Fan, Z. Shen, Y. Zhao, Y. Sun et al., Heterojunction TiO_2@$TiOF_2$ nanosheets as superior anode materials for sodium-ion batteries. J. Mater. Chem. A **9**(9), 5720-5729 (2021). https://doi.org/10.1039/d0ta12340d

[S3] Y. Zhang, Y. Huang, V. Srot, P.A. Aken, J. Maier et al., Enhanced pseudo-capacitive contributions to high-performance sodium storage in TiO_2/C nanofibers via double effects of sulfur modification. Nano-Micro Lett. **12**, 165 (2020). https://doi.org/10.1007/s40820-020-00506-1

[S4] H. Li, J. Lang, S. Lei, J. Chen, K. Wang et al., A high-performance sodium-ion hybrid capacitor constructed by metal-organic framework-derived anode and cathode materials. Adv. Funct. Mater. **28**(30), 1800757 (2018). https://doi.org/10.1002/adfm.201800757

[S5] M. Kang, Y. Wu, X. Huang, K. Zhou, Z. Huang et al., Engineering of a TiO_2 anode toward a record high initial coulombic efficiency enabling high-performance low-temperature Na-ion hybrid capacitors. J. Mater. Chem. A **6**(45), 22840-22850 (2018). https://doi.org/10.1039/c8ta07127f

[S6] W. Feng, R.R. Maça, V. Etacheri, High-energy-density sodium-ion hybrid capacitors enabled by interface-engineered hierarchical TiO_2 nanosheet anodes. ACS Appl. Mater. Interfaces **12**(4), 4443-4453 (2020). https://doi.org/10.1021/acsami.9b17775

[S7] Y. Fang, Y. Zhang, C. Miao, K. Zhu, Y. Chen et al., Mxene-derived defect-rich TiO_2@rGO as high-rate anodes for full na ion batteries and capacitors. Nano-Micro Lett. **12**, 128 (2020). https://doi.org/10.1007/s40820-020-00471-9

[S8] J.Y. Hwang, H.L. Du, B.N. Yun, M.G. Jeong, J.S. Kim et al., Carbon-free TiO_2 microspheres as anode materials for sodium ion batteries. ACS Energy Lett. **4**(2), 494-501 (2019). https://doi.org/10.1021/acsenergylett.8b02510

[S9] Z. Chen, L. Xu, Q. Chen, P. Hu, Z. Liu et al., Spray-pyrolysis-assisted synthesis of yolk@shell anatase with rich oxygen vacancies for efficient sodium storage. J. Mater. Chem. A **7**(12), 6740-6746 (2019). https://doi.org/10.1039/c8ta11440d

[S10] S. Luo, T. Yuan, L. Soule, J. Ruan, Y. Zhao et al., Enhanced ionic/electronic transport in nano-TiO_2/sheared cnt composite electrode for Na^+ insertion-based hybrid ion-capacitors. Adv. Funct. Mater. **30**(5), 1908309 (2020). https://doi.org/10.1002/adfm.201908309

[S11] Y. Zhang, X. He, J. Tang, J. Jiang, X. Ji et al., Sulfur-doped TiO_2 anchored on a large-area carbon sheet as a high-performance anode for sodium-ion battery. ACS Appl. Mater. Interfaces **11**(47), 44170-44178 (2019). https://doi.org/10.1021/acsami.9b14597

[S12] X. Xu, B. Chen, J. Hu, B. Sun, X. Liang et al., Heterostructured TiO_2 spheres with tunable interiors and shells toward improved packing density and pseudocapacitive sodium storage. Adv. Mater. **31**(46), e1904589 (2019). https://doi.org/10.1002/adma.201904589

[S13] W. Song, H. Zhao, L. Wang, S. Liu, Z. Li, Co-doping nitrogen/sulfur through a solid-

state reaction to enhance the electrochemical performance of anatase TiO_2 nanoparticles as a sodium-ion battery anode. ChemElectroChem **5**(2), 316-321 (2018). https://doi.org/10.1002/celc.201701015

[S14] J. Ni, S. Fu, Y. Yuan, L. Ma, Y. Jiang et al., Boosting sodium storage in TiO_2 nanotube arrays through surface phosphorylation. Adv. Mater. **30**(6), 1704337 (2018). https://doi.org/10.1002/adma.201704337

[S15] R.R. Maça, D.C. Juárez, M.C. Rodríguez, V. Etacheri, Nanointerface-driven pseudocapacitance tuning of TiO_2 nanosheet anodes for high-rate, ultralong-life and enhanced capacity sodium-ion batteries. Chem. Eng. J. **391**, 123598 (2020). https://doi.org/10.1016/j.cej.2019.123598

[S16] J. Feng, Y. Dong, Y. Yan, W. Zhao, T. Yang et al., Extended lattice space of TiO_2 hollow nanocubes for improved sodium storage. Chem. Eng. J. **373**, 565-571 (2019). https://doi.org/10.1016/j.cej.2019.05.065

[S17] X. Wang, L. Qi, H. Wang, Anatase TiO_2 as a Na^+-storage anode active material for dual-ion batteries. ACS Appl. Mater. Interfaces **11**(33), 30453-30459 (2019). https://doi.org/10.1021/acsami.9b09703

[S18] X. Guo, J. Zhang, J. Song, W. Wu, H. Liu et al., MXene encapsulated titanium oxide nanospheres for ultra-stable and fast sodium storage. Energy Storage Mater. **14**, 306-313 (2018). https://doi.org/10.1016/j.ensm.2018.05.010
